# ANALYZING THE INFLUENCE OF MIDLIFE EMPLOYMENT TRAJECTORIES ON COGNITIVE AGING IN CHILE

**DOI:** 10.1093/geroni/igae098.3600

**Published:** 2024-12-31

**Authors:** Magdalena Delaporte, Jere Behrman

**Affiliations:** University of Pennsylvania, Philadelphia, Pennsylvania, United States; University of Pennsylvania, Philadelphia, Pennsylvania, United States

## Abstract

As the global population ages, understanding the factors contributing to healthy aging becomes increasingly crucial. This paper delves into the relationship between mid-life employment trajectories and later-life cognition, utilizing a Chilean longitudinal dataset. Employing the Group-Based Trajectory Model (GBTM), distinct employment trajectories for males and females between 1980 and 2016 are estimated. Subsequently, we link these trajectories to cognitive function scores to investigate the associations between individual trajectories and cognitive function in later life. Preliminary findings reveal significant associations between certain cognitive domains and labor force participation trajectories. We find four distinct labor force participation trajectories for men and women. Results show that men that have a high probability of continued employment demonstrate better cognitive outcomes, including orientation, memory, attention, and executive function. For women, a higher likelihood of working during between ages 30 to 60 correlates with improved performance in delayed memory, attention, and executive function. These results provide valuable insights for policymakers and practitioners seeking to enhance cognitive well-being among older adults.

